# Larotrectinib versus infigratinib for adult patients with both glioma and tyrosine kinase alterations after failure of initial therapies: Efficacy and safety analysis

**DOI:** 10.1016/j.clinsp.2024.100329

**Published:** 2024-02-07

**Authors:** Yufang Chen, Jian Ma, Qianqian Gao, Yu Gai, Yichi Sun, Meihua Wang

**Affiliations:** a Department of Pathology, Changzhou Tumor Hospital, Changzhou, Jiangsu, China; b Department of Oncology, Changzhou Tumor Hospital, Changzhou, Jiangsu, China

**Keywords:** Glioma, Hyperphosphatemia, Infigratinib, Larotrectinib, Tyrosine kinase alteration

## Abstract

**Objectives:**

To compare the efficacy and safety of larotrectinib with those of infigratinib in adult glioma patients with tyrosine kinase alterations.

**Methods:**

Patients received oral infigratinib 125 mg (IN cohort, *n* = 125) or oral larotrectinib (LB cohort, *n* = 105) until unacceptable toxicity or disease progression.

**Results:**

Duration of treatment was longer in the LB cohort than in the IN cohort (8 [9.5–6.25] months vs. 5.5 [6–5.25] months, *p* < 0.0001). Patients with partial responses (*p* = 0.0424) and overall survival (*p* = 0.03) were higher in the IN cohort than those in the LB cohort. The number of patients with disease progression was higher in the LB cohort (*p* = 0.0015). All the patients reported diarrhea, fatigue, vomiting, constipation, and decreased appetite. Patients in the IN cohort reported hyperphosphatemia, hyperlipasemia, stomatitis, dry skin, alopecia, dyspepsia, onycholysis, palmar-plantar erythrodysesthesia, nail disorders, and dry eyes. Patients in the LB cohort reported upper respiratory tract infections, pyrexia, cough, anemia, bacterial/viral infections, conjunctivitis, urinary tract infections, headaches, ataxia, dizziness, and muscle tremors. A total of 30 (24 %) and 40 (38 %) patients from the IN and the LB cohorts died at the follow-up of 18 months (*p* = 0.03). Patients who received bevacizumab initial therapy had higher overall survival (*p* = 0.048).

**Conclusions:**

Infigratinib has higher efficacy and overall survival than larotrectinib but has higher adverse effects in the management of both glioma and tyrosine kinase alterations after failure of initial therapies. Initial bevacizumab therapy is associated with a higher overall survival.

## Highlights

• Tyrosine kinase domains with genomic alterations have oncogenic potential.
• Higher efficacy for infigratinib than larotrectinib.
• Infigratinib has a higher overall survival than larotrectinib.
• Infigratinib has higher adverse effects than larotrectinib.
• Bevacizumab initial therapy has a higher overall survival.

## Introduction

Gliomas are a type of neuroepithelial tissue^1^ that is about 2 % of all types of occurring cancers (rare diseases).^2^ Glioblastomas are aggressive and lethal types of gliomas.^3^ The tyrosine kinase domains of genomic alterations in gliomas demonstrate oncogenic potential.^4^ Tyrosine kinase domains are available with fibroblast growth factor receptors^5^ and play a vital role in neurotrophic tyrosine receptor kinase gene encoding.^6^ Tyrosine kinase alterations have a vital role in the development of glioma.^5^^,^^6^ Limited literature is available on the mechanism of action of tyrosine kinase alterations responsible for the development of glioma.^7^

Larotrectinib (tropomyosin receptor kinase inhibitor) monotherapy^6^ and infigratinib (tyrosine kinase inhibitor) monotherapy^5^ are both effective in the treatment of gliomas with tyrosine kinase alterations in adult patients. However, to date, larotrectinib monotherapy has not been compared with infigratinib monotherapy for the treatment of glioma, where tyrosine kinase alterations occur in the development of glioma.

The objectives of this retrospective study were to compare progression-free survival, overall survival, treatment response, and adverse effects between adult patients with glioma with tyrosine kinase alterations who received larotrectinib and those who received infigratinib.

## Materials and methods

### Ethics approval and consent to participate

The designed protocol was prepared by the authors and approved by the Human Ethics Committee of Changzhou Tumor Hospital (Approval number CJ20220224 dated January 10, 2016) and the Chinese Society of Clinical Oncology. As this was a retrospective study, informed consent of patients and/or their legally authorized person(s) was waived by the human ethics committee of the Changzhou Tumor Hospital. The study follows the laws of China and the v2008 Declarations of Helinski.

### Inclusion criteria

Adult glioma patients with pathologically confirmed tyrosine kinase alterations after failure of initial therapy were included in the study.

### Exclusion criteria

Patients who required anticonvulsant drugs (e.g., carbamazepine, phenobarbital, and phenytoin) were excluded from the study (because of strong inducers of CYP3A4). In addition, patients with abnormal calcium and/or phosphate homeostasis, neurological instability, history of corneal/keratopathy, or retinal disorders were excluded from the study. Patients with a history of sensitivity to larotrectinib and infigratinib were excluded from this study.

### Cohorts

A total of 125 patients received oral infigratinib 125 mg once daily on days 1–21 of each 28-day cycle until unacceptable toxicities or disease progression (IN cohort).^5^ The patients were recommended a low-phosphate diet. In cases of hyperphosphatemia (plasma inorganic phosphate level > 4.5 mg/dL), oral phosphate binder(s) were provided to patients. A total of 105 patients received 100 mg twice daily of oral larotrectinib until unacceptable toxicities or disease progression (LB cohort).^6^ The Common Terminology Criteria for Adverse Events, version 4.03 was used to define unacceptable toxicities. Disease progression was evaluated using magnetic resonance imaging and pathology.

### ECOG performance status

ECOG (Eastern Cooperative Oncology Group) performance status was graded as 0 ‒ Fully active; 1 ‒ Unable to perform strenuous activities; 2 ‒ Capable of all self-care activities but dependent on others to carry out normal activities; 3 ‒ Capable of performing limited self-care activities; 4 ‒ Completely disabled.

### Treatment response evaluation

Magnetic Resonance Imaging (MRI) was performed every 2 months. RECIST 1.1 criteria^8^ was used to evaluate treatment response. Oncologists, in assistance with radiologists, evaluated the treatment response. The responses are listed in Table 1.^9^

**Table 1:** Treatment response evaluation.

Different types of responses	Criteria
Complete response	Complete invisible of all advanced measurables and unmeasurable diseases.
Partial response	50 % or more decrease of all advanced measurables and unmeasurable diseases. That is sustained for at least 4-weeks, with no new lesions.
Progression	25 % or more increase in all advanced measurables and unmeasurable diseases.
Stable disease	Not qualified for partial response and no progression of the disease.

Oncologists in assistance with radiologists have evaluated treatment response.

Magnetic resonance imaging scans and pathology were preferred for evaluation of response.

### Survival

#### Progression-free survival

Survival of patient without any progression of disease after treatment(s).

#### Overall survival

The detection of disease(s) to death was considered the overall survival of the patient.

New disease lesions or disease progression were detected using magnetic resonance imaging scans and pathology.

### Adverse effects

The Common Terminology Criteria for Adverse Events, version 4.03, was used to evaluate adverse effects during the treatment(s) and follow-up period.^10^

#### Hyperphosphatemia

A plasma inorganic phosphate level of more than 4.5 mg/dL was considered hyperphosphatemia. Blood pathology was performed to detect the plasma inorganic phosphate levels.

### Statistical analysis

InStat 3.01 (GraphPad Software, San Diego, CA, USA) was used for the statistical analysis. All results were considered significant if the *p*-value was less than 0.05. Categorical, continuous normal, and continuous non-normal variables are presented as frequencies (percentages), mean±Standard Deviation (SD), and median (Q3–Q1), respectively. Fisher's exact test or the Chi-Square test (χ^2^-test) was performed for categorical variables. Kolmogorov and Smirnov tests were performed to check the normality of continuous variables. The Mann-Whitney test was used for the statistical analysis of non-normal continuous variables. Multivariate analysis was used for statistical analysis of the evaluation of independent parameters (treatment, gender, ethnicity, ECOG status, and initial therapies) for higher overall survival at a 95 % Confidence Interval (95 % CI).

## Results

### Study population

**Fig. 1:**
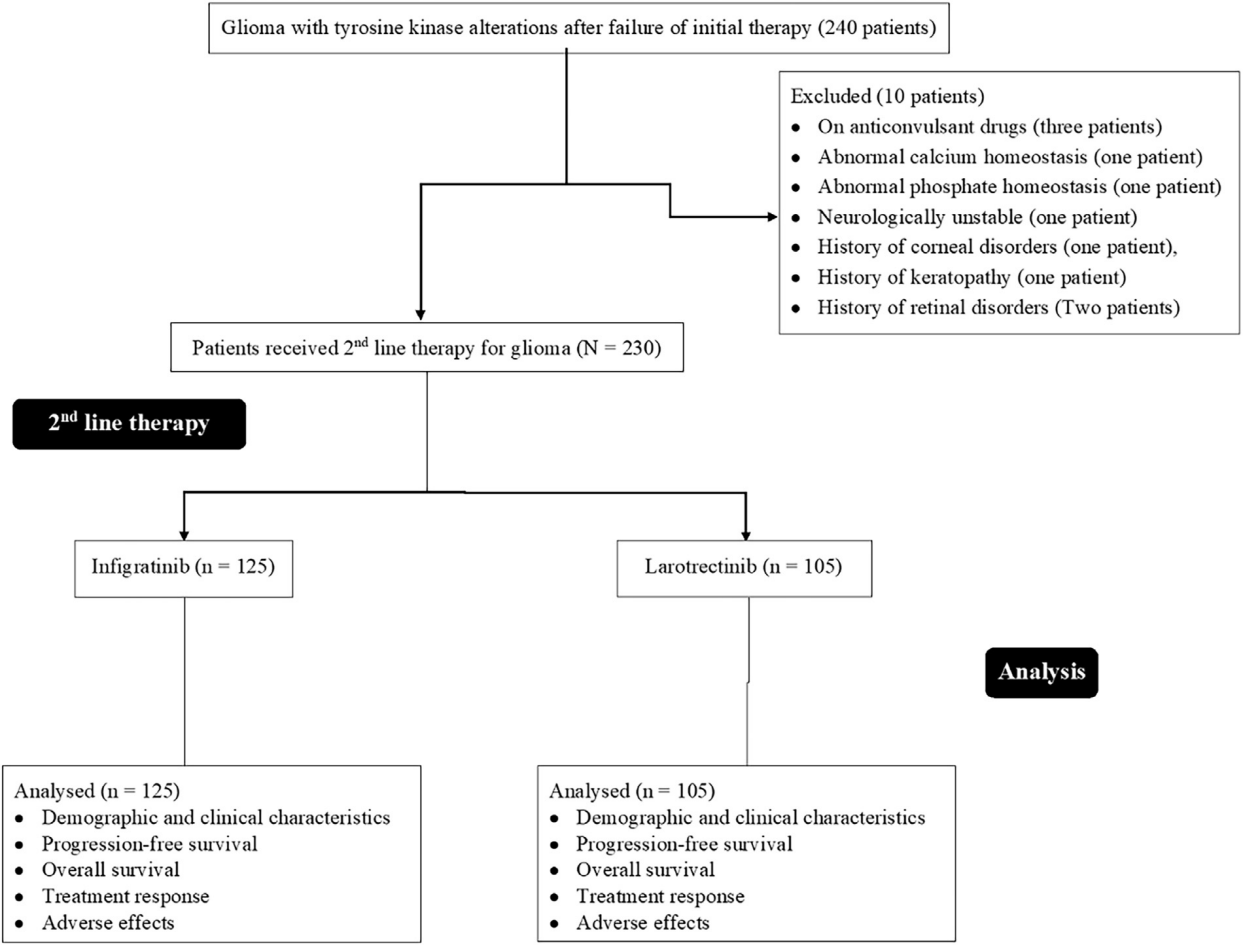
The retrospective flow chart.

From January 15, 2016, to December 17, 2018, 240 adult patients with glioma with tyrosine kinase alterations after the failure of initial therapy were available at the Changzhou Tumor Hospital, Changzhou, Jiangsu, China, and the referring hospitals. Among them (240 glioma patients), three patients were on anticonvulsant drugs, one patient had abnormal calcium homeostasis, one patient had abnormal phosphate homeostasis, one patient was neurologically unstable, one patient had a history of corneal disorders, one patient had a history of keratopathy, and two patients had a history of retinal disorders. Therefore, these patients (10 glioma patients) were excluded from the study. The medical records of progression-free survival, overall survival, treatment response, and adverse effects of 230 patients with glioma with tyrosine kinase alterations after the failure of initial therapy were included in the analysis. A retrospective flowchart of the study is presented in Fig. 1.

### Demographic and clinical parameters

The number of male patients was higher than that of female patients. More than 50 % of patients had ECOG performance status 1. There were no significant differences in sex, age, ethnicity, ECOG performance status, initial therapies (i.e., failure) between cohorts before the start of second-line treatment for glioma (previous treatments received including radiation), the time frame of diagnosis, medicine administration, extent of surgery, prior history of cancer, and genetic background of participants. The details of the demographic and clinical parameters of the enrolled patients before the start of second-line treatment for glioma are reported in Table 2.

**Table 2:** Demographic and clinical parameters of enrolled patients before the start of second-line treatment for glioma.

Parameters		Total	Cohorts		Comparisons between cohorts			
			IN	LB				
Treatment(s)		2nd line	Infigratinib	Larotrectinib				
Numbers of patients		230	125	105	p-value	95 % CI	Df	Test value
Gender	Male	135 (59)	70 (56)	65 (62)	0.4404 (χ^2^-test)	0.7071 to 1.134	1	0.5952
	Female	95 (41)	55 (44)	40 (38)				
Age (years)		52 (57–44)	52 (57–44)	52 (56.5–43)	0.5211 (Mann-Whitney test)	N/A	N/A	6239.5
Ethnicity	Han Chinese	214 (93)	117 (94)	97 (92)	0.7292 (χ^2^-test for Trend)	N/A	1	0.1199
	Mongolian	14 (6)	7 (5)	7 (7)				
	Tibetan	2 (1)	1 (1)	1 (1)				
ECOG performance status	0	55 (24)	30 (24)	25 (24)	0.9799 (χ^2^-test for independence)	N/A	2	0.0451
	1	140 (56)	70 (56)	60 (57)				
	2	45 (20)	25 (20)	20 (19)				
Initial Therapies	Radiotherapy	25 (11)	15 (12)	10 (10)	0.7456 (χ^2^-test for independence)	N/A	3	1.231
	Antineoplastic therapy	60 (26)	35 (28)	25 (24)				
	Bevacizumab	85 (37)	45 (36)	40 (38)				
	Temozolomide	60 (26)	30 (24)	30 (28)				

Categorial variables are depicted as frequencies (percentages). Continuous non-normal variables are depicted as median (Q3–Q1).

CI, Confidence Interval (using the approximation of Katz.); Df, Degree of freedom; N/A, Not Applicable; ECOG, Eastern Cooperative Oncology Group (0: Fully active; 1: Unable to do strenuous activities; 2: Capable of all self-care but dependent on others to carry out normal activities).

Test value (χ^2^ value for χ^2^-test, Mann-Whitney U-statistic for Mann-Whitney test).

### Treatment response evaluation

The duration of treatment was higher in the LB cohort than in the IN cohort (8 [9.5–6.25] months vs. 5.5 [6–5.25] months, *p* < 0.0001, Mann–Whitney test, Mann–Whitney U-statistic = 1811). Patients with a complete response and stable disease were statistically similar between the cohorts. However, the number of patients with partial responses was higher in the IN cohort. The number of patients with disease progression was higher in the LB cohort. The details of the treatment response evaluation after the termination of 2nd line treatment are reported in Table 3.

**Table 3:** Treatment response evaluation after the termination of 2nd line treatment.

Parameters	Total	Cohorts		Comparisons between cohorts			
		IN	LB				
Treatment(s)	2^nd^ line	Infigratinib	Larotrectinib				
Numbers of patients	230	125	105	*p*-value	95 % CI	Df	Test value
Complete response	22 (10)	13 (10)	9 (9)	0.6611 (Fisher exact test)	0.7581 to 1.588	N/A	1.097
Partial response	62 (27)	41 (33)	21 (20)	0.0424 (χ^2^-test with Yate's correction)	1.047 to 1.671	1	4.121
Progression	69 (30)	26 (21)	43 (41)	0.0015 (χ^2^-test with Yate's correction)	0.4418 to 0.8500	1	10.097
Stable disease	77 (33)	45 (36)	32 (30)	0.4569 (χ^2^-test with Yate's correction)	0.8777 to 1.423	1	0.5535

Variables are depicted as frequencies (percentages).

RECIST 1.1 criteria were used for the evaluation of treatment response.

Oncologists in assistance with radiologists have evaluated treatment response.

CI, Confidence Interval (using the approximation of Katz.); Df, Degree of freedom; N/A, Not Applicable.

Test value (χ^2^ value or relative risk value for χ^2^-test Fisher exact test).

### Survival

From the start of treatment and after the termination of 2nd line treatment in a follow-up of 18 months, a total of 14 (11 %) and 10 (10 %) patients from the IN and the LB cohorts had progression-free survival. Progression-free survival of patients was the same between cohorts from the start of treatment, during treatment, and in followed-up of 18 months after termination of 2nd line treatment (*p* = 0.999, Fisher’s exact test, 95 % CI: 0.6270 to 1.471, Relative Risk = 0.9603). From the start of treatment, during treatment, and at the follow-up of 18 months a total of 30 (24 %) and 40 (38 %) patients from the IN and the LB cohorts died. overall survival of patients in the IN cohort was higher than that of the LB cohort (*p* = 0.03, χ^2^-test with Yates correction, degree of freedom: 1, 95 % CI: 0.5350 to 0.9738, χ^2^-value: 4.71).

### Adverse effects

Patients in the IN cohort reported hyperphosphatemia, diarrhea, fatigue, vomiting, hyperlipidemia, stomatitis, dry skin, alopecia, decreased appetite, dyspepsia, onycholysis, palmar-plantar erythrodysesthesia, constipation, nail disorder, and dry eyes during 2nd line treatment and 18-months of followed-up. Patients in the LB cohort reported diarrhea, fatigue, vomiting, decreased appetite, constipation, upper respiratory tract infection, pyrexia, cough, anemia, bacterial and/or viral infection, conjunctivitis, urinary tract infection, headache, ataxia, dizziness, and muscle tremor during 2nd line treatment and 18-months of followed-up. The details of the adverse effects during 2nd line treatment and 18 months of follow-up are reported in Table 4.

**Table 4:** Adverse effects during 2nd line treatment and 18-months of followed-up.

Parameters	Cohorts		Comparisons between cohorts			
	IN	LB				
Treatment(s)	Infigratinib	Larotrectinib				
Numbers of patients	125	105	*p*-value	95 % CI	Df	Test value
Hyperphosphatemia	94 (75)^a^	0 (0)	<0.0001 (Fisher exact test)	3.220 to 5.978	N/A	4.378
Diarrhea	20 (16)^a^	60 (57)^b^	<0.0001 (χ^2^-test with Yate's correction)	0.2409 to 0.5295	1	40.788
Fatigue	30 (24)^a^	55 (52)^b^	<0.0001 (χ^2^-test with Yate's correction)	0.3946 to 0.7354	1	18.529
Vomiting	65 (52)^a^	60 (57)^b^	0.5176 (χ^2^-test with Yate's correction)	0.7185 to 1.153	1	0.4187
Hyperlipasemia	15 (12)^a^	0 (0)	<0.0001 (Fisher exact test)	1.715 to 2.227	N/A	1.955
Stomatitis	14 (11)^a^	0 (0)	0.0001 (Fisher exact test)	1.709 to 2.216	N/A	1.946
Dry skin	13 (10)^a^	0 (0)	0.0003 (Fisher exact test)	1.703 to 2.204	N/A	1.938
Alopecia	22 (18)^a^	0 (0)	<0.0001 (Fisher exact test)	1.760 to 2.316	N/A	2.019
Decreased appetite	18 (14)^a^	18 (17)^b^	0.5893 (Fisher exact test)	0.6385 to 1.287	N/A	0.9065
Dyspepsia	9 (7)^a^	0(0)	0.0043 (Fisher exact test)	1.681 to 2.160	N/A	1.905
Onycholysis	8 (6)^a^	0 (0)	0.0085 (Fisher exact test)	1.675 to 2.149	N/A	1.897
Palmar-plantar erythrodysesthesia	7 (6)^a^	0 (0)	0.0166 (Fisher exact test)	1.670 to 2.139	N/A	1.89
Constipation	12 (10)^a^	32 (30)^b^	<0.0001 (Fisher exact test)	0.2733 to 0.7374	N/A	0.4489
Nail disorder	11 (9)^a^	0(0)	0.0011 (Fisher exact test)	1.692 to 2.181	N/A	1.921
Dry eye	8 (6)^a^	0(0)	0.0085 (Fisher exact test)	1.675 to 2.149	N/A	1.897
Upper respiratory tract infection	0 (0)	55 (54)^b^	<0.0001 (Fisher exact test)	-Infinity to Infinity	N/A	0
Pyrexia	0 (0)	35 (33)^b^	<0.0001 (Fisher exact test)	-Infinity to Infinity	N/A	0
Cough	0(0)	31 (30)^b^	<0.0001 (Fisher exact test)	-Infinity to Infinity	N/A	0
Anemia	0 (0)	32 (30)^b^	<0.0001 (Fisher exact test)	-Infinity to Infinity	N/A	0
Bacterial/Viral infection	0 (0)	22 (21)^b^	<0.0001 (Fisher exact test)	-Infinity to Infinity	N/A	0
Conjunctivitis	0 (0)	19 (18)^b^	<0.0001 (Fisher exact test)	-Infinity to Infinity	N/A	0
Urinary tract infection	0 (0)	15 (14)^b^	<0.0001 (Fisher exact test)	-Infinity to Infinity	N/A	0
Headache	0 (0)	21 (20)^b^	<0.0001 (Fisher exact test)	-Infinity to Infinity	N/A	0
Ataxia	0 (0)	7 (7)^b^	0.0037 (Fisher exact test)	-Infinity to Infinity	N/A	0
Dizziness	0 (0)	5 (3)^b^	0.0188 (Fisher exact test)	-Infinity to Infinity	N/A	0
Muscle tremor	0 (0)	4 (4)	0.0421 (Fisher exact test)	-Infinity to Infinity	N/A	0

Variables are depicted as frequencies (percentages). The Common Terminology Criteria for Adverse Events, version 4.03 was used for the evaluation of adverse effects.

CI, Confidence Interval (using the approximation of Katz.); Df, Degree of freedom; N/A, Not Applicable.

Test value (χ^2^ value or relative risk value for χ^2^-test Fisher exact test).

a Infigratinib-emergent adverse effects.

b Larotrectinib-emergent adverse effects. Hyperphosphatemia: > 4.5 mg/dL plasma inorganic phosphate level.

### Independent parameter

Patients who received bevacizumab initial therapy had higher overall survival (*p* = 0.048, Odds Ratio: 1.0241, 95 % CI: 1.0011 to 1.3211, multivariate analysis).

## Discussions

Patients with partial response were higher in the IN cohort, and patients with progression were higher in the LB cohort after termination of 2nd line treatment. The poor blood-brain barrier penetration of Larotrectinib^6^^,^^11^ is responsible for the higher progression of disease in patients in the LB cohort. Infigratinib can penetrate the central nervous system^5^ and is responsible for a higher partial response in patients in the IN cohort. Infigratinib has higher efficacy than larotrectinib in adult patients with both glioma and tyrosine kinase alterations after the failure of initial therapies.

Progression-free survival was the same, but overall survival was higher among adult patients with glioma with tyrosine kinase alterations after the failure of initial therapies who received infigratinib than among those who received larotrectinib. Partial response was higher and disease progression was lower among adult patients with glioma with tyrosine kinase alterations after the failure of initial therapies who received infigratinib, which would lead to an increase in overall survival of patients, but these were not enough for progression-free survival of patients. Infigratinib increases the overall survival of adult patients with both glioma and tyrosine kinase alterations after the failure of initial therapies.

Larotrectinib-emergent adverse effects were higher than infigratinib-emergent adverse effects. Larotrectinib has a favorable safety profile in both adult and pediatric patients.^6^^,^^12^ The infigratinib-emergent adverse effects observed in the current study are consistent with those in clinical trials.^5^^,^^13^ Infigratinib has more adverse effects than larotrectinib in the management of both glioma and tyrosine kinase alterations after failure of initial therapies.

Initial bevacizumab therapy is associated with a higher overall survival. The results of the independent parameters for overall survival in the current study are consistent with those of the phase II study.^5^ A combination of bevacizumab with infigratinib or larotrectinib could result in higher overall survival of patients with both glioma and tyrosine kinase alterations.

Performing chi square or Mann-Whitney tests are inadequate for evaluating the overall survival in cancer treatment. In almost all clinical evaluations of overall survival, median survival times, and cancer-related survival rates, the statistical analysis for comparison between treatment options is performed by Kaplan Meier estimator curves. The possible justifications for the same are Kaplan Meier estimator curves used for predictions of overall survival and progression-free survival. However, in the current study the authors have absolute parameters for overall survival and progression-free survival because of the 18 months of followed-up period of data. As because of the availability of absolute values, the authors have evaluated overall survival and progression-free survival using Fisher’s exact test instead of Kaplan–Meier estimator curves.

The study proposed to perform a clinical evaluation for efficacy and safety analysis of larotrectinib or infigratinib therapies for patients with both glioma and tyrosine kinase alterations. However, there are certain limitations of this study, for example, its retrospective design and the lack of randomized trials. The study did not report or discuss the efficacy of infigratinib and larotrectinib in recurrent disease conditions. The absence of WHO 2021 CNS tumor classification^14^ applied to what seems to be collectively referred to as “glioma”, and thereby it is not possible to draw conclusions regarding the efficacy of these two drugs. What constitutes glioma is currently understood to represent a rather vast array of tumors that must be evaluated based on their molecular characteristics to draw any valid conclusions.

## Conclusions

Infigratinib has a higher efficacy than larotrectinib in adult patients with both glioma and tyrosine kinase alterations after the failure of initial therapies. Infigratinib increases the overall survival compared to larotrectinib in adult patients with both glioma and tyrosine kinase alterations. Infigratinib has more adverse effects than larotrectinib in the management of adult patients with both glioma and tyrosine kinase alterations after the failure of initial therapies. A combination of bevacizumab with infigratinib or larotrectinib could result in higher overall survival of patients with glioma.

## Availability of data and materials

The datasets used and analyzed during the current study are available from the corresponding author upon reasonable request.

## Authors’ contributions

All the authors have read and approved the manuscript for publication. YC and JM were project administrators who contributed equally to the conceptualization, formal analysis, supervision, resources, methodology, validation, and literature review of the study. QG contributed to the investigation, resources, conceptualization, visualization, formal analysis, methodology, and literature review. YG contributed resources, formal analysis, conceptualization, methodology, data curation, and literature review. YS contributed to the resources, supervision, formal analysis, data curation, and literature review. MW contributed to the resources, funding acquisition, formal analysis, methodology, and literature review of the study and drafted and edited the manuscript for intellectual content. All authors agree to be accountable for all aspects of this study, ensuring its integrity and accuracy. YC and JM confirmed the authenticity of the raw data.

## Funding

This study was supported by the Changzhou Sci & Tech Program, China (Grant no. CJ20220224).

## Conflicts of interest

The authors declare no conflicts of interest or any competing interest**s** regarding the results and/or discussions reported in this research.
